# Familial hypomagnesaemia with hypercalciuria and nephrocalcinosis (FHHNC): Compound heterozygous mutation in the claudin 16 *(CLDN16*) gene

**DOI:** 10.1186/1471-2369-9-12

**Published:** 2008-09-24

**Authors:** Geeta Hampson, Martin A Konrad, John Scoble

**Affiliations:** 1 Department of Chemical Pathology, St Thomas Hospital, London, UK; 2 Department of Pediatric Nephrology, University Children's Hospital, Waldeyerstrasse 22, Munster, Germany; 3 Renal Unit, Guy's Hospital, London, UK

## Abstract

**Background:**

Familial hypomagnesaemia with hypercalciuria and nephrocalcinosis (FHHNC) is an autosomal recessive disorder of renal calcium and magnesium wasting frequently complicated by progressive chronic renal failure in childhood or adolescence.

**Methods:**

A 7 year old boy was investigated following the findings of marked renal insufficiency and nephrocalcinosis in his 18-month old sister. He too was found to have extensive nephrocalcinosis with increased fractional excretion of magnesium: 12.4% (<4%) and hypercalciuria: 5.7 mmol (< 2.5/24 hours). He had renal impairment, partial distal renal tubular acidosis and defective urinary concentrating ability. Therapy with thiazide diuretics and magnesium supplements failed to halt the progression of the disorder. Both children subsequently underwent renal transplantation. Both children's parents are unaffected and there is one unaffected sibling.

**Results:**

Mutation analysis revealed 2 heterozygous mutations in the claudin 16 gene *(CLDN16*) in both affected siblings; one missense mutation in exon 4: C646T which results in an amino acid change Arg216Cys in the second extracellular loop of *CLDN16 *and loss of function of the protein and a donor splice site mutation which changes intron 4 consensus splice site from 'GT' to 'TT' resulting in decreased splice efficiency and the formation of a truncated protein with loss of 64 amino acids in the second extracellular loop.

**Conclusion:**

The mutations in *CLDN16 *in this kindred affect the second extra-cellular loop of claudin 16. The clinical course and molecular findings suggest complete loss of function of the protein in the 2 affected cases and highlight the case for molecular diagnosis in individuals with FHHNC.

## Background

Hereditary renal tubular disorders associated with impaired renal conservation of salt, calcium and magnesium comprise a wide range of clinical symptoms, metabolic/biochemical abnormalities and underlying genetic defects. Mutations in several genes involved in renal electrolyte transport have been identified as the cause of many of the tubular disorders such as Bartter's syndrome, Gitelman syndrome, isolated renal magnesium loss or familial hypomagnesaemia with hypercalciuria and nephrocalcinosis (FHHNC) [1-6].

FHHNC is an autosomal recessive disorder of renal calcium and magnesium wasting often complicated by, as yet, unexplained progressive chronic renal failure during childhood or adolescence. Patients usually present with recurrent urinary tract infections, polyuria/polydipsia, nephrolithiasis/calcinosis. Loss of function mutation of the claudin 16 (*CLDN16*) gene have been identified as the underlying genetic defect in the majority of patients affected with FHHNC. *CLDN16 *encodes claudin 16, a tight junction protein which is expressed in the thick ascending limb (TAL) of the loop of Henle [5].

We report 2 heterozygous mutations of this gene in 2 siblings with this disorder and provide information about their phenotypic presentation and clinical course. We also present relevant data on their parents and unaffected sibling.

## Methods

### Case report

The index case, 2 siblings and his parents underwent clinical, biochemical and genetic analyses. The index case is the oldest sibling and aged 7 at the time of hospital admission for diagnostic work-up as his younger sister had been diagnosed with nephrocalcinosis and hypomagnesaemia, aged 18 months. She was first thought to have renal problems at the age of 18 months, having been investigated for failure to thrive from the age of 10 months. She had recurrent urinary tract infections and was shown to have reflux into one ureter and impaired renal function. She did not have hypertension at the time of presentation and did not require anti-hypertensive treatment until later when she was started on Atenolol 25 mg/daily. Her blood pressure was subsequently well controlled on this treatment. Unlike his younger sister, the index case had not had any acute presentations necessitating hospital admissions earlier. He had been generally well but closer questioning revealed a history of polyuria/polydipsia, unexplained feverish illnesses, possibly due to frequent urinary tract infections since early childhood. In contrast to his younger sibling, he had marked hypertension. Physical examination revealed arterial hypertension (B.P 170/130 mmHg). His height was 126.6 cm (75^th ^centile) and his weight 23.5 kg (50^th ^centile). Examination of his heart, lungs, abdomen and nervous system was unremarkable. There was no evidence of ocular abnormalities or hearing impairment. They are Caucasians and there was no history of parental consanguinity.

Preliminary laboratory investigations in the index case revealed renal impairment (sodium: 142 mmol/L, potassium: 3.6 mmol/L, urea: 7.1 mmol/L, creatinine: 93 mcmol/L, GFR: 51 mls/min/1.73 m^2^). Serum calcium was normal (2.38 mmol/L), magnesium was low (0.63 mmol/L, reference range 0.7–1.0 mmol/l), phosphate was 1.52 mmol/L. Parathyroid hormone (PTH) was elevated, 600 ng/L (normal range: < 120). Urinary analyses revealed hypercalciuria, 5.67 mmol/24 hours (normal range: < 2.5/24 hours). Fractional excretion rate of magnesium was elevated: 12.4% (< 4%). Water deprivation test indicated a defect in urinary concentrating ability. Ammonium chloride loading test revealed a distal defect in urinary acidification. Abdominal plain radiographs revealed bilateral nephrocalcinosis and large kidneys. Based on the clinical and laboratory findings a diagnosis of FHHNC was made. Treatment was started with magnesium supplementation and chlorothiazide. The hypercalciuria was resistant to chlorothiazide which was later discontinued as it aggravated hypomagnesaemia. He was also treated with propanolol 40 mg three times daily and hydralazine 3.5 mg three times daily. His blood pressure was satisfactory on these medications and came down to 100/60. The index case's growth rate was not compromised to the same extent as his younger sibling. Treatment, however failed to prevent the progression of renal failure in both children. They eventually underwent renal transplantation. The younger sibling required renal transplantation at an earlier age as the decline in GFR was more rapid in her case compared to her older sibling whose renal functions were better preserved. Her GFR was 27 mls/min at age 5, having declined from 32 ml/min in 2 years. Her pre-transplant results at age 9 years showed normal serum electrolytes, urea: 35.2 mmol/L, creatinine: 454 mcmol/L, calcium: 1.69 mmol/L, phosphate: 1.69 mmol/L, magnesium: 0.73 mmol/L. The affected younger sibling received a deceased donor transplant. In contrast the index case's GFR remained stable at 40 ml/min during his early teenage years. However his renal function declined further in his late teens and he received a live donor kidney in young adulthood from his father.

All subjects, including the index case and his affected younger sibling who are now adults aged 33 and 28 years respectively gave their consent. The parents and the unaffected sibling were also screened. The parents and unaffected child's biochemical results were normal. They had no history of renal calculi. Urinary calcium, magnesium and phosphate excretion was normal. Blood was also obtained from all members of the family for genetic analyses after informed consent. This had been approved by the Research Ethics Committee of Guy's Hospital, UK. Consent for publication was obtained from the patients.

### Molecular studies

DNA was extracted from peripheral blood using standard protocols. *CLDN16 *mutation screening was performed by single-strand conformation polymorphism analysis [7]. Amplified products of the coding sequence (exons 1 to 5) were separated on a denaturing polyacrylamide gel by electrophoresis. The exons with the conformational variants compared to controls were then sequenced directly. Mutational analysis of the *CLDN16 *gene was carried out by direct sequencing of both strands of the coding region and the adjacent exon/intron boundaries following polymerase chain reaction (PCR) amplification of genomic DNA with flanking intronic primers. All primer sequences are available upon request. DNA sequencing was performed using the fluorimetric method (Big-Dye Terminator Cycle sequencing kit; ABI 3700 DNA sequencer, Applied Biosystems, Foster City, Ca, USA) according to the manufacturer's protocol.

## Results

**Figure 1:**
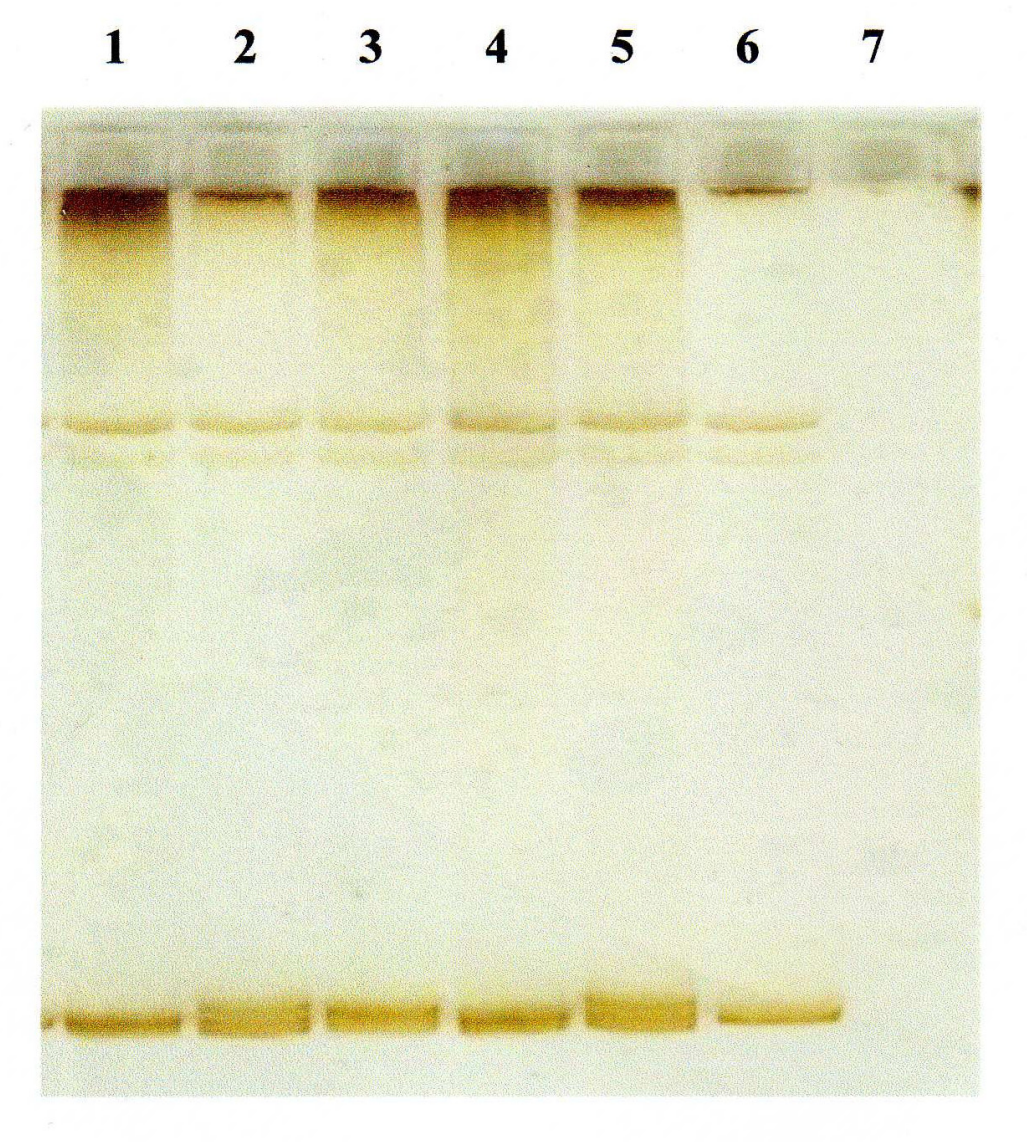
**Single strand polymorphism analysis of the amplified products of exon 4 of *CLDN16 *gene showing the conformational variants.** Heterozygous carriers: Lanes 1: Unaffected sibling, Lane 3: Mother, Lane 4: Father. Affected cases: Lanes 2 and 5. Control: Lane 6. Blank: Lane 7.

**Figure 2:**
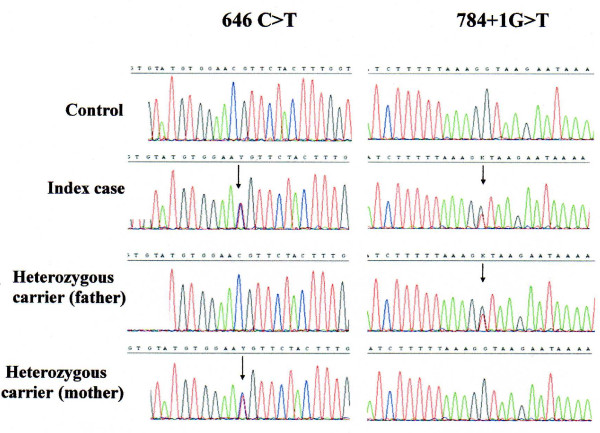
***CLDN16***** sequence analysis of the index case and his parents showing the heterozygous mutations.**

Two types of conformational variants were identified in exon 4 in all family members compared to controls. This is illustrated in Figure 1. Lanes 2 and 5 represent the affected children. Lanes 1, 3, 4 correspond to the unaffected family members who are heterozygote carriers. Direct sequencing of exon 4 revealed 2 heterozygous mutations; one mis-sense mutation 646 C> T (inherited from the maternal allele) and a second novel mutation which is a splice site mutation 784 +1 G>T (inherited from the paternal allele) as shown in Figure 2 (Genbank Acc number NM_006580). The unaffected sibling was a heterozygous carrier of the paternal mutation (784 +1 G>T). The missense mutation is located in the second extracellular loop of claudin 16 and leads to an amino acid change from Arginine to Cysteine. This missense mutation has been shown following expression analysis to result in complete loss of function of the protein [8]. The 784 +1 G>T splice mutation results in a change in the intronic consensus splice site such that 'GT' is changed to 'TT'. This leads to a 224-fold decrease in splice efficiency as calculated from the automated splice site analysis. The mutation leads to skipping of exon 4 with a loss of 64 amino acids in the second transmembrane domain and is likely to lead also to loss of function.

## Discussion

Familial hypomagnesaemia with hypercalciuria and nephrocalcinosis is a rare autosomal recessive renal tubular disorder that is frequently complicated by chronic renal failure in early childhood or adolescence [5,9,10]. Both cases exhibited the most commonly observed renal complications. They did not have any extra-renal manifestations. The clinical observations and clearance studies point to impaired reabsorption of magnesium and calcium in the TAL of the loop of Henle as the primary defect. The TAL of the loop of Henle plays a predominant role in the renal reabsorption of divalent cations which mainly pass via paracellular flux [11]. Treatment failed to prevent progression of the disease in the 2 cases presented here as previously reported for other patients [9]. The heterozygous carriers had no evidence of hypercalciuria and/or nephrolithiasis. Renal transplantation in both affected individuals corrected the abnormal renal magnesium and calcium handling. It is unclear why renal failure occurs in FHHNC and has been linked to the nephrocalcinosis. However in other renal tubular disorders where extensive medullary nephrocalcinosis occurs such as in ante-natal Bartter syndrome, progressive renal failure to the same extent as that seen in FHHNC is not observed [12]. This may be related to the different molecular defects in these 2 tubulopathies.

Genotyping of the index case and the family members revealed 2 heterozygous mutations of *CLDN16*. The *CLDN16 *gene codes for a protein which is almost exclusively expressed in the TAL of the loop of Henle and spans the lipid bilayer 4 times with 2 extracellular loops [13]. Over 20 different mutations have been identified to date. The majority of mutations (67%) are single-nucleotide changes which occur in the first extra-cellular loop [9]. We detected 2 mutations in exon 4 which codes for the second extracellular loop. Very few mutations have been previously described in the second extracellular loop. A recently reported mutation in exon 4 of the *CLDN16 *gene, located in the 4^th ^transmembrane domain, was shown to be been associated with extreme short stature which was not a clinical feature in our cases [14]. The missense mutation described here leads to a change from arginine to cysteine. In general cysteine residues are known to lead to conformational changes or alterations in trafficking or ion selectivity. This mutation has previously been reported in a Japanese patient and a substitution of the same amino acid to threonine has also been reported in an Algerian family with FHHNC [15,9]. This may indicate a mutation cluster at this site in the second extra-cellular loop and confirms the physiological importance of the second loop. Although previous mis-sense mutations have been described in exon 4, this is the first splice site mutation reported in this region of the *CLDN16 *gene. The splice site mutation leads to a significant reduction in splice efficiency and the formation of a truncated second extra-cellular loop. The 2 mutations described here resulted in classical FHHNC with severe and rapid deterioration of renal function. This may be explained by the complete loss of function of the protein. Indeed expression of the 646 C> T mutation has been shown to lead to an almost complete loss of function [8]. It is also likely that the splice-site mutation results in loss of function of the protein. The clinical course in patients bearing two mutations with complete loss of function (including also stop-, frameshift- and splice-site mutations) is significantly worse compared to patients with at least one mutation with residual function (partial loss of function). Significantly more patients with two complete loss of function mutations have ESRD and require renal replacement therapy earlier in life compared to patients with residual function which may delay the progression of renal failure [8].

The observations of a rather close intra-familial concordance with respect to the clinical course therefore supports the important role of distinct *CLDN16 *genotypes in the severity and rapidity of decline in renal function in FHHNC [9]. Hence differences in disease severity between families may be attributed to the location of the mutation within the gene. Indeed, a milder clinical course which consists of hypercalciuria during childhood with conserved kidney function has been described in 2 families with a homozygous mutation in the C-terminal domain of the protein; T233R. This point mutation affects the interaction of claudin 16 with the tight junction scaffolding protein ZO-1 leading to disruption of localisation of the protein to the tight junctions in renal epithelial cells with accumulation in the lysosomes [16]. Other tight junction proteins have also been implicated in FHHNC. Recently mutations in claudin 19 (*CLDN19*) have been demonstrated in several families with the classical renal phenotype of FHHNC [17]. However the affected members of these families also had severe visual impairment consistent with the expression of this tight-junction protein not only in the kidney but also in the retina.

The heterozygote carriers of the family in the present report did not exhibit any disturbances in the renal handling of calcium and magnesium and did not have any clinical evidence of nephrolithiasis as in some previously reported families [18,19]. Thus the decision for the father to be a kidney donor was acceptable and carried no increased risk of stone formation for the live related donor.

## Conclusion

In conclusion the missense and splice site heterozygous mutations in this family with classical presentation of FHHNC affecting the second extracellular loop of *CLDN16 *provides evidence of the functional importance of the second loop in the paracellular transport of magnesium and calcium. In addition the severity of the clinical presentation in the 2 affected cases points to complete loss of function of claudin 16 and emphasises the importance of molecular diagnosis in this condition.

## Competing interests

The authors declare that they have no competing interests.

## Authors' contributions

GH made a substantial contribution to the acquisition and analysis/interpretation of data on this family and in drafting the manuscript. MK made a substantial contribution to the analysis and interpretation of data and in revising the manuscript critically for important intellectual content. JS made a substantial contribution to the diagnosis and acquisition of data and in revising the manuscript critically for important intellectual content.

## Pre-publication history

The pre-publication history for this paper can be accessed here:


